# Electro-osmotic Treatment of Dredged Sediment by Different Power Supply Modes: Energy Consumption and Electro-osmotic Transport Volume

**DOI:** 10.1038/s41598-019-49050-y

**Published:** 2019-09-03

**Authors:** Lingwei Zheng, Xiada Zhu, Xinyu Xie, Jinzhu Li

**Affiliations:** 1 Research Center of Coastal and Urban Geotechnical Engineering, Zhejiang University, Hangzhou, 310058 China; 2 Engineering Research Center of Urban Underground Development of Zhejiang Province, Hangzhou, 310058 China; 3 Ningbo Institute of Technology, Zhejiang University, Ningbo, 315100 China

**Keywords:** Electrochemistry, Civil engineering

## Abstract

Laboratory model tests were conducted in constant-voltage mode and constant-current mode for the one-dimensional electro-osmotic treatment of dredged sediment, with an approximately consistent initial electric power. The voltage, current, drainage rate, electro-osmotic transport volume, and energy consumption coefficient during the electro-osmotic process were measured and calculated. After treatment, the final soil moisture at designated positions in the test samples was measured to investigate the effects of different power supply modes. Further, the divergent phenomena observed with constant voltage and constant current were discussed. Based on an analysis of the measured energy consumption coefficients with time, we obtained a linear relationship between the applied/equivalent voltage and energy consumption coefficient. Furthermore, the electro-osmotic processes are divided into four stages by equal drainage quantity to obtain the energy consumption and electro-osmotic transport volume under different working conditions. The results reveal that the energy consumption of electro-osmosis is mainly determined by the applied voltage or the equivalent voltage for dredged sediment, while the value of electro-osmotic transport volume depends mainly on the change in soil water content rather than power supply modes. The drainage rate in constant-current mode was observed to be relatively steady, maintaining an approximately constant rate until the soil moisture was dramatically reduced. In other words, constant-current mode shows the advantages of being powerful and persistent in electro-osmotic treatment.

## Introduction

Since the first application of electro-osmosis on soft soil reinforcement conducted by Casagrande^1^ as an electrochemical treatment, electro-osmosis has been widely used in sludge dewatering, tailings remediation, soil reinforcement, etc. Unlike mechanical dewatering^2,3^ or vacuum preloading^4,5^, electro-osmotic dewatering can effectively treat soils with a low permeability coefficient, such as dredged sediment. Nevertheless, due to the higher energy consumption and lower efficiency in the late stage of treatment, the electro-osmosis method has some limitations in the practical engineering of soil reinforcement.

The soil particles are negatively charged, while the part of the double layer that is contained in the adjoining liquid is positively charged. Therefore, by applying an electric field, the ions with a positive charge are moved from the anode to the cathode carrying the associated water molecules with them^1^; meanwhile, electrochemical reactions occur. Thus, the soil reinforcement is attributed to two main processes: the discharge of pore water and multiple chemical reactions. The electrochemical treatment of soil includes the electrolysis of water, electrode corrosion, ion migration, cementation, etc. These chemical reactions are expected to alter the geotechnical properties of soils^6^. Chang *et al*.^7^ added CaCl_2_ solution to kaolin during electro-osmosis, accompanied with a pozzolanic reaction, and found that the shear strength of kaolin was significantly affected by the concentration of the solution and pH value. To improve the efficiency of electro-osmosis and reduce the cost, researchers have mainly studied the electrode arrangement used in electro-osmotic treatment, the power supply type, combined treatment methods, and supplementary chemical solutions.

The effect of the electrode plays a key role in the electro-osmotic process; by optimizing the electrode arrangement^8,9^, researchers have found that the reinforcement effect can be improved considerably. Most researchers employ a constant power output to ensure the regularity of monitoring data. Micic *et al*.^10^ and Ibeid *et al*.^11^ studied the effect of intermittent current conditions on electro-osmosis and noted that intermittent current can improve the utilization of electric energy and make the soil reinforcement more uniform. Liu *et al*.^12^ studied the effect of stepwise loading on electro-osmosis and noted that a reasonable stepwise voltage loading scheme can reduce the power consumption while improving the electro-osmotic drainage and treatment efficiency. The direct current (DC) supply used for soil electro-osmosis can be obtained from an alternating current (AC) supply by using a rectifier and a filter. Chien and Ou^13^ proposed converting the minus wave to a plus wave by using half-wave rectification and full-wave rectification during electro-osmosis, which reduced the cost of the power supply. Streche *et al*.^14^ conducted two types of experiments using soil that was artificially contaminated and revealed that, with an approximately threefold increase in the quantity of treated soils, the specific energy consumption decreased.

Electrode arrangement, stepwise loading voltages, intermittent currents, and other means of controlling power supply with variable voltages do not change the mechanism by which electric energy is converted to water flow. However, there has been little study to date on constant-current conditions and the essential distinction between constant voltage and constant current. In this paper, laboratory tests of soil electro-osmosis are carried out in constant-voltage mode and constant-current mode in one-to-one correspondence under an approximately consistent initial electric power.

## Experimental Design

### Material properties

**Figure 1:**
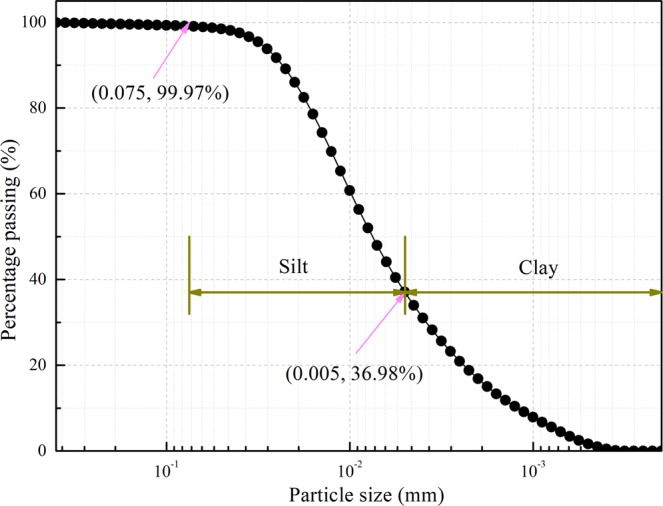
Grain size distribution curve of intact dredged sediment.

The dredged sediment treated in the tests was obtained from a depth of 0.5 metres below the channel of a river in Ningbo, China, which is fluid-plastic and grey-black with stink. The particle size distribution curve of the dredged sediment was obtained using a laser particle analyser (Malvern Mastersizer 2000, produced by Malvern Instruments Ltd., UK), as shown in Fig. 1. The content of silt, which possessed a particle size in the range of 0.075~0.005 mm, was 62.99%; the content of clay, which exhibited a particle size of less than 0.005 mm, was 36.98%. The test results show that the dredged sediment is mainly composed of fine-grained soil. The typical properties of the dredged sediment are summarized in Table 1.

**Table 1:** Properties of the dredged sediment.

Property	Value
Specific gravity, *Gs*	2.52
Initial soil moisture, *w* (%)	70
Density, *ρ* (g/cm^3^)	1.64
Plastic limit, *w*_P_ (%)	27
Liquid limit, *w*_L_ (%)	35
Organic matter content (%)	3–4

Before the tests, the dredged sediment was dried and ground into powder. The powder was then remoulded with the target moisture content, which was 2.0 times greater than its liquid limit. To ensure the uniformity of the specimen, the remoulded dredged sediment was placed into the test chambers layer by layer, which was vibrated mechanically to eliminate the air bubbles, and then allowed to stand for 24 h in preparation of the specimen.

### Apparatus and procedure

The DC power apparatus with a programmable logic controller (PLC) used in the tests was produced by Beijing HSPY Technology Co. Ltd. and was capable of constant-voltage mode output with real-time current display and constant-current mode output with real-time voltage display. For constant-current mode, the programmable logic controller (PLC) was utilized to maintain the set output current value through real-time voltage adjustments; therefore, as the load resistance increased (decreased), the output voltage increased (decreased).

**Figure 2:**
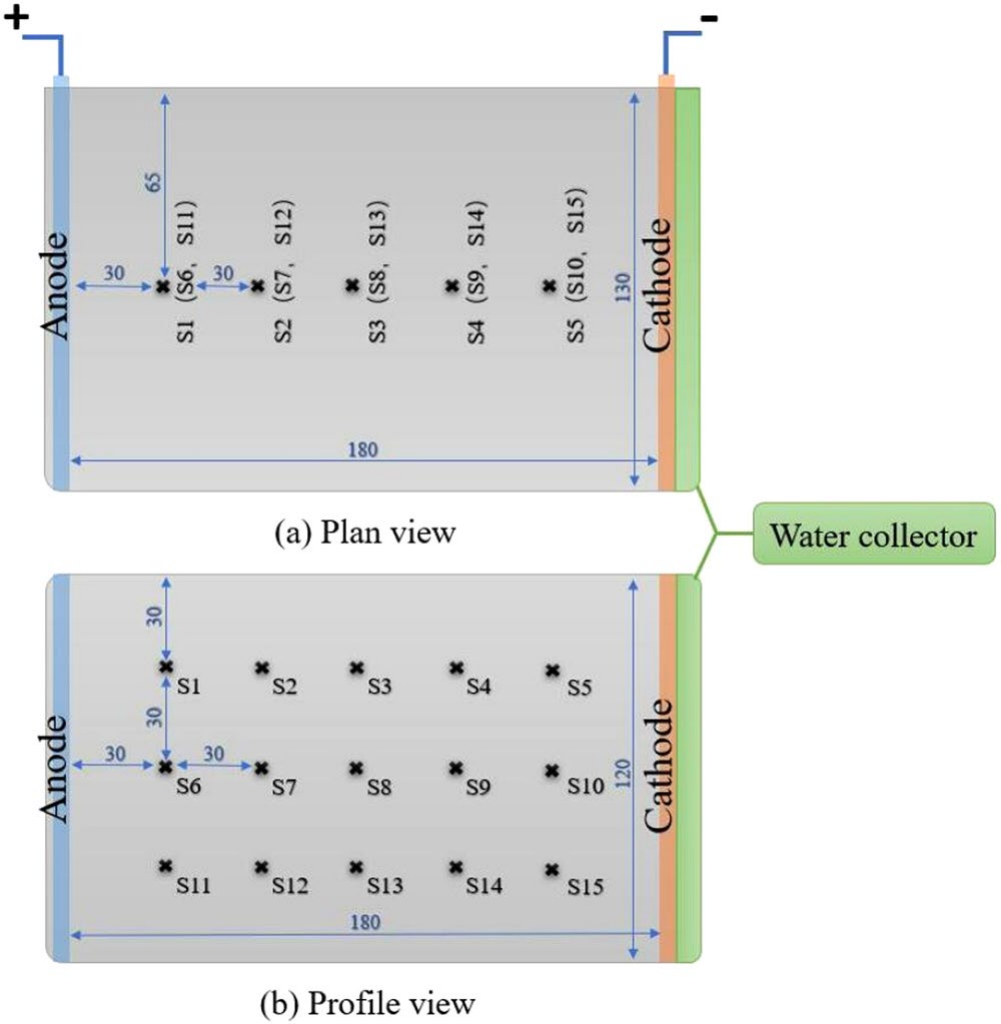
Model test chamber for the electro-osmotic treatment of the dredged sediment (unit: mm).

The one-dimensional electro-osmotic consolidation apparatus mainly included a test chamber, an anode, and a cathode outlet. The internal dimensions of the test chamber used to hold the dredged sediment and anode/cathode electrodes were 180 mm × 120 mm × 130 mm. The dimensions of the plate-shaped electrodes were 120 mm × 130 mm × 3 mm, as shown in Fig. 2.

The anodes and cathodes were all made of 304 stainless steel; specifically, the plate-shaped cathodes were drilled with holes by laser-drilling and covered with a geotextile filter layer. After the anode/cathode electrodes had been installed, the soil samples were compactly filled into the chamber layer by layer. When an electrical field was applied with a constant voltage or current, the pore water of the dredged sediment moved from the anode towards the cathode and then flowed into the water collector underneath the cathode. The voltage, current and cumulative drainage were recorded every 30 minutes during the tests. After treatment, the water moisture of the soil samples was measured at various positions, which are indicated by crosses in Fig. 2.

### Power control

Previous studies conventionally set a constant voltage to study other influencing factors, such as the electrode material^15^, electrode arrangement^9^, and polarity reversal^16^. To study the effect and determine the functional mechanism of electro-osmotic dewatering in constant-voltage mode and constant-current mode, we adopted the following experimental scheme, as shown in Table 2.

**Table 2:** Summary of electro-osmotic tests.

Test	Power supply control	Initial voltage (V)	Initial current (A)	Initial power (W)
V1	Constant voltage	9.5	0.05	0.475
V2	Constant voltage	16.5	0.1	1.68
V3	Constant voltage	23.5	0.15	3.525
A1	Constant current	9.25	0.05	0.4625
A2	Constant current	16.06	0.1	1.606
A3	Constant current	22.75	0.15	3.4125

During testing, the initial output power was purposely kept consistent for each pair of conditions (e.g., V1 and A1) as the controlling factor. Tests V1 and A1, tests V2 and A2, and tests V3 and A3 each had approximately consistent initial electric power; the deviation in each parallel group was controlled within 5%. Furthermore, the electrode gap in all tests was maintained equal to facilitate comparative analysis.

## Results and Analysis

### Voltage and current

**Figure 3:**
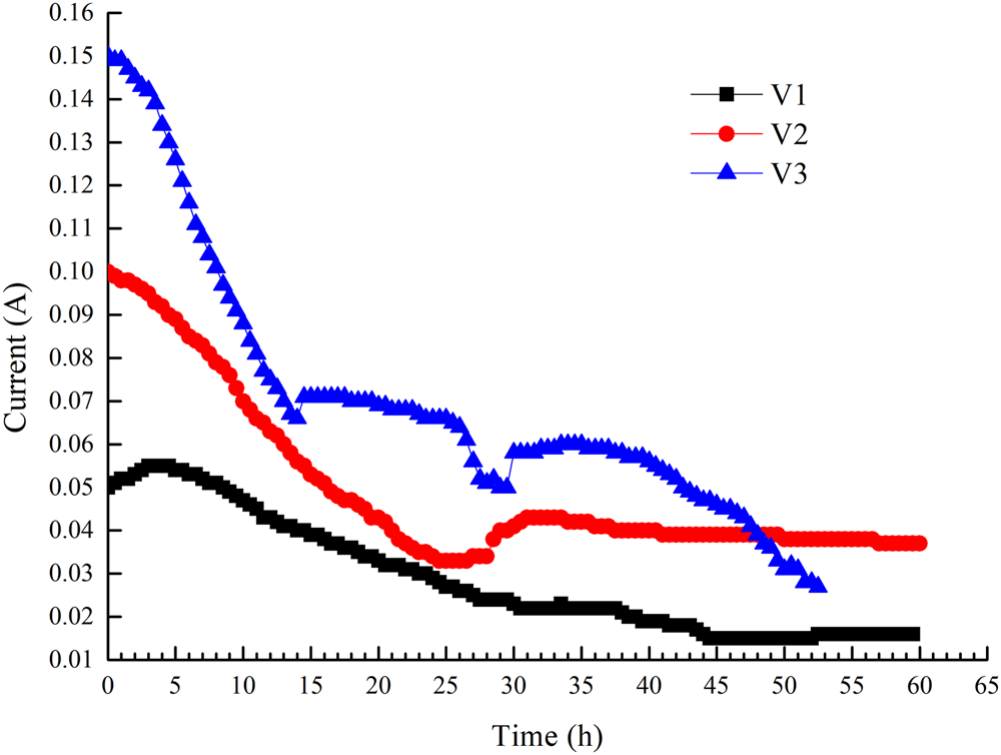
Variations in electric current with time under constant voltage.

**Figure 4:**
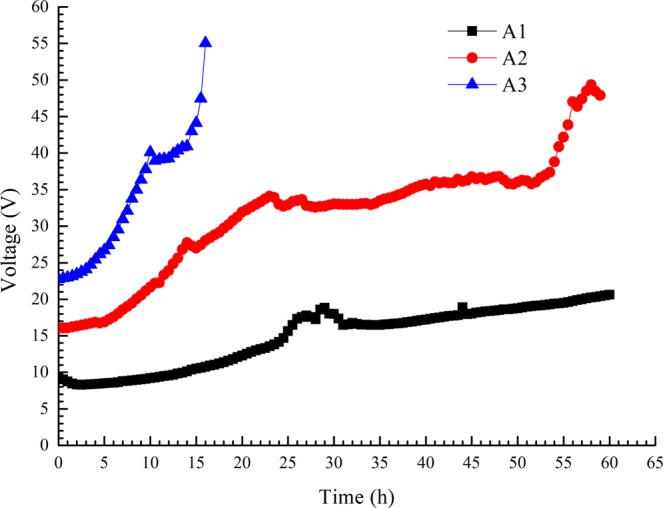
Variations in electric voltage with time under constant current.

As the electro-osmotic process continued, the electric current decreased in constant-voltage mode, while the electric voltage increased in constant-current mode. A more apparent trend was observed with a higher constant voltage or current, as shown in Figs 3 and 4. We terminated test A3 to avoid power failure when the output voltage exceeded the rated voltage.

This relationship between current and voltage mainly depends on the increase in resistance caused by electro-osmotic dewatering. As one factor, soil near the anode region lost moisture rapidly and formed a hard shell as gas escaped, which greatly increased the interface resistance^17^. Additionally, current attenuation is related to the transmission path in soil. Three transmission paths are considered to be relevant to electric current in soil: pore water, soil particles, and soil-water interfaces^18^. When the soil moisture is higher, electric current mainly transmits through the pore water; when the soil moisture is lower, electric current mainly transmits through the soil-water interfaces. As the electro-osmotic process continues, uneven moisture loss from the soil always leads to the nonlinear development of electric resistance.

### Drainage rate and final soil moisture

**Figure 5:**
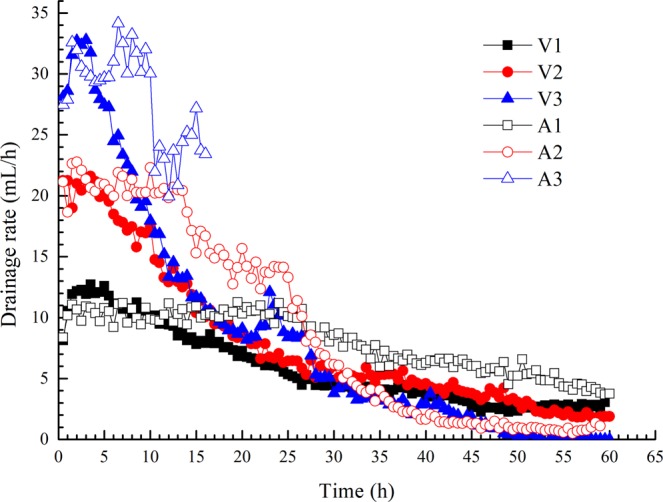
Variations in drainage rate with time.

The variations in drainage rate with time are shown in Fig. 5. Comparing the two groups of tests, the drainage rate in constant-current mode was observed to be relatively steady, maintaining an approximately constant rate until the soil moisture was dramatically reduced. In other words, constant-current mode shows the advantages of being powerful and persistent in electro-osmotic treatment.

**Figure 6:**
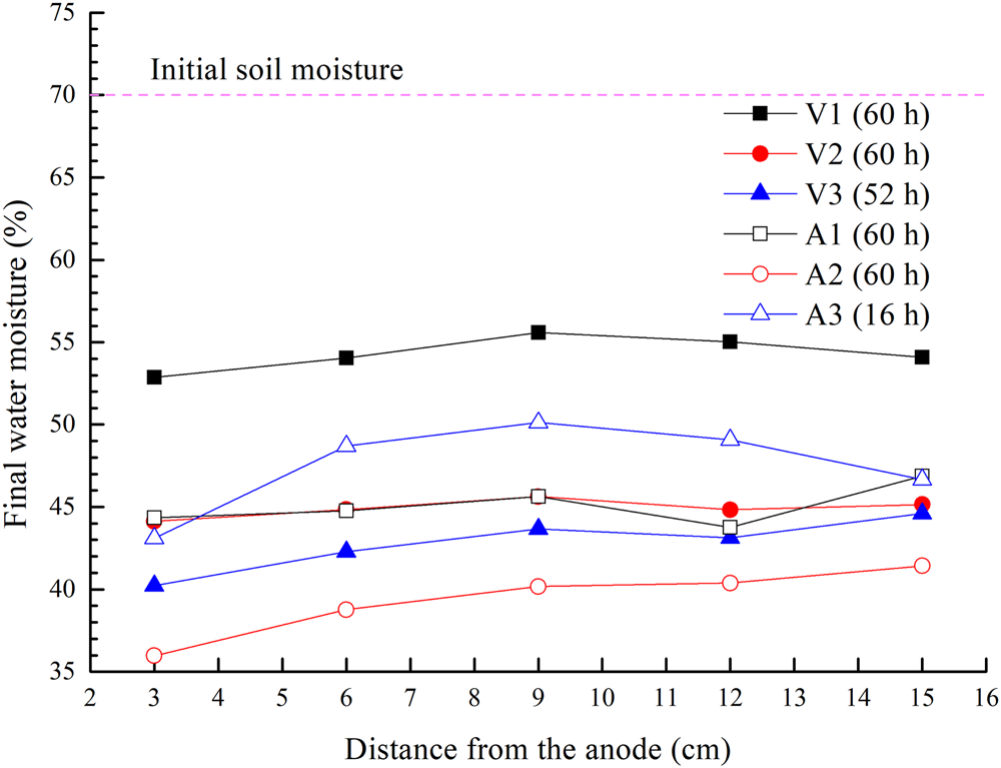
Final moistures in the soil at different positions.

The discrepancy between the initial and final values is believed to be illustrative of the dewatering effects of different electrodes. The soil moistures before and after electro-osmotic treatment were measured. The initial soil moisture in all tests was 70%, and the final soil moistures at different positions are shown in Fig. 6. As the pore water in the soil flowed from the anode to cathode during electro-osmosis, the soil moisture in the anode region was generally lower than that in the cathode region for each group; the final water moisture increased with the distance from the anode. The final moisture in test A1 was almost equal to that of test V2, while the trends in moisture in tests V3 and A2 were similar. The results indicate that the effect of treatment in constant-voltage mode is similar to that in constant-current mode for a given type of soil. Incidentally, for the episode with the power supply cessation, Fig. 6 shows the water content of test A3 is higher than that of the other ones, due to the duration (15 h) of electro-osmosis being shorter than that of the other tests (60 h).

### Output power

**Figure 7:**
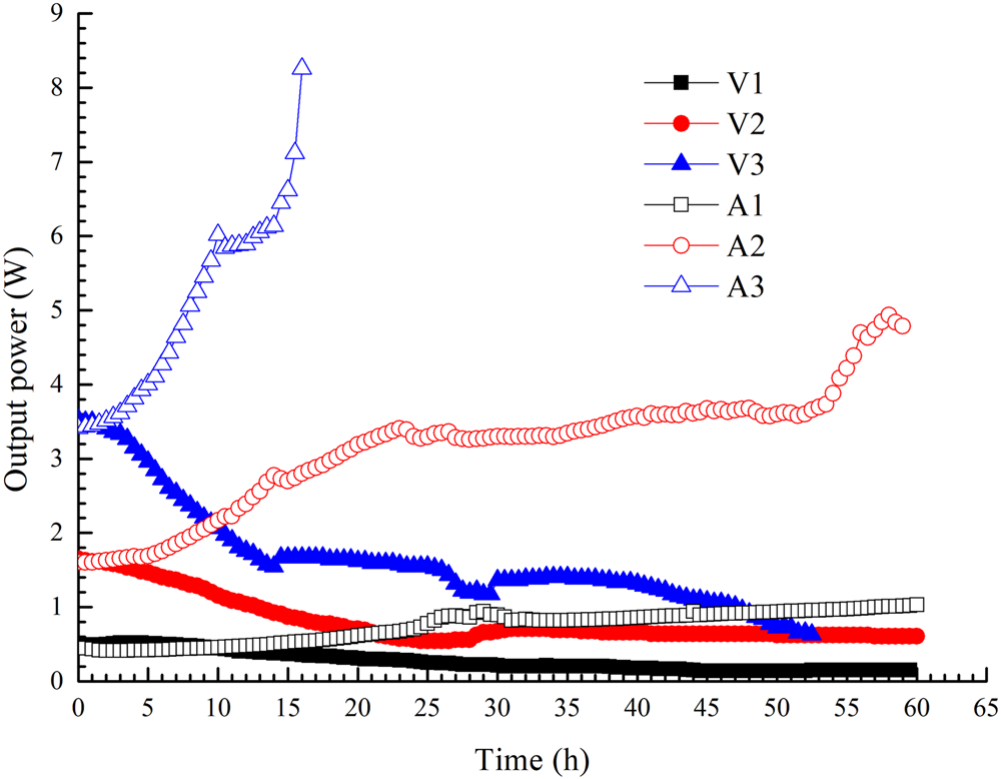
Variations in output power with time.

Figure 7 shows the variations in output power with time during testing. Unlike the behaviour observed in constant-voltage mode, the output power increased with time in constant-current mode. The initial output power determines the subsequent power variation in the electro-osmotic process: the higher the former is, the more obvious the latter. As the power-time curves show, the output power in tests V3 and A3 was dramatically transformed during electro-osmosis, while the output power in tests V1 and A1 remained in a relatively low range.

### Measured electro-osmotic transport volume

The electro-osmotic transport volume is defined as the volume of water transported by a unit charge^19^, which reflects the utilization rate of electric energy. In practical engineering, this quantity can be calculated by the ratio of electro-osmotic drainage rate to electric current:

1 $$W=\frac{{q}_{{\rm{e}}}}{I}$$

where *q*_e_ is the electro-osmotic drainage rate in mL·h^−1^ and *I* is the electric current in A.

Under the conditions of different electrode materials or electric potential gradients, the relationship between the electro-osmotic drainage rate and the electric current is linear for a given type of soil^20,21^. Thus, the electro-osmotic transport volume is constant under ideal conditions.

According to Darcy’s law (Eq. (2)) and Ohm’s law (Eq. (3)), the electro-osmotic transport volume can be expressed as Eq. (4).

2 $${q}_{{\rm{e}}}={k}_{{\rm{e}}}{i}_{{\rm{e}}}A$$

3 $$I={\sigma }_{{\rm{e}}}{i}_{{\rm{e}}}A$$

4 $$W={k}_{{\rm{e}}}/{\sigma }_{{\rm{e}}}$$

where *k*_e_ is the electro-osmotic permeability coefficient in cm^2^·h^−1^·V^−1^, *i*_e_ is the electric potential gradient in V·cm^−1^, *A* is the cross-sectional area of the soil in cm^2^, and *σ*_e_ is the electrical conductivity in S·cm^−1^.

Most researchers assume that the electro-osmotic transport volume *W* in the process of electro-osmosis has a constant value for a given type of soil. During electro-osmosis in ideal conditions, the electro-osmotic permeability coefficient *k*_e_ and the electrical conductivity *σ*_e_ of the channel dredged sediment will simultaneously decrease. Furthermore, the initial electro-osmosis transport volume is not sensitive to changes in soil moisture. When the soil moisture is lower than a certain limit value, the electro-osmotic permeability and electro-osmotic transport volume will be greatly reduced due to the generation of soil cracks and shrinkage^22^.

**Figure 8:**
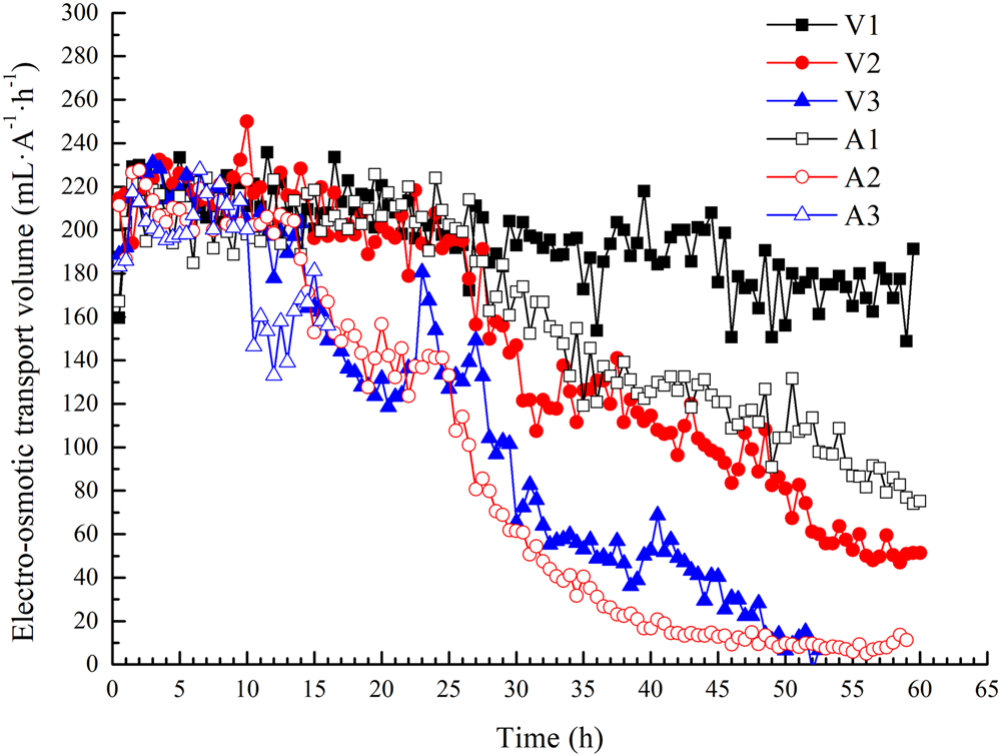
Measured electro-osmotic transport volume with time.

Previous studies have commonly studied the electro-osmotic transport volume in constant-voltage mode. In this work, electro-osmotic transport volume data obtained under constant-voltage and constant-current conditions were compared and analysed. Figure 8 shows the measured electro-osmotic transport volume under different working conditions.

In the early stage (0 ~ 10 h) of electro-osmosis, the value of the electro-osmotic transport volume *W* was approximately 210 mL·A^−1^·h^−1^ in all tests. Evidently, the electro-osmotic transport volume for a given type of soil is independent of the working conditions until the properties of the soil have been substantially changed. At a certain point, the measured electro-osmotic transport volume will significantly decrease with the shrinking and cracking of soil due to soil dehydration. In particular, higher power loading (voltage or current) conditions can cause this point to be reached earlier. Therefore, we adopted the data from the early stages of the tests for further analysis.

### Energy consumption coefficient

As one of the key problems in practical engineering for electro-osmosis in soft soil, the energy consumption coefficient *C* is used to characterize the electrical energy consumed per unit dewatering volume, broadly defined as:

5 $$C=\frac{{\int }_{{t}_{1}}^{{t}_{2}}I(t){\rm{\Delta }}E{\rm{d}}t}{{\int }_{{t}_{1}}^{{t}_{2}}{q}_{{\rm{e}}}(t){\rm{d}}t}$$

where *I*(*t*) is the electric current at time *t* in A; Δ*E* is the electric potential difference, i.e., practical applied voltage in constant-voltage mode in V; and *q*_e_(*t*) is the electro-osmotic drainage rate at time *t* in h.

Assuming that the electro-osmotic drainage rate is constant in the electro-osmotic process, the electro-osmotic drainage rate can be defined as:

6 $${q}_{{\rm{e}}}(t)=W\,I(t)$$

Combining the energy consumption coefficient expression (Eq. (5)) and the electro-osmotic drainage rate expression (Eq. (6)), the electro-osmotic energy consumption coefficient can be rewritten as:

7 $$C=\frac{{\rm{\Delta }}E{\int }_{{t}_{1}}^{{t}_{2}}I(t){\rm{d}}t}{W{\int }_{{t}_{1}}^{{t}_{2}}I(t){\rm{d}}t}=\frac{{\rm{\Delta }}E}{W}$$

The power supply in the circuit provides both the voltage and current. Thus, the power supply can be represented as either a voltage source or current source. For a given internal resistance and load of the power supply, the voltage source and current source can be equivalently transformed. In the tests, all electrode gaps were equal. In constant-voltage mode, the electric potential difference Δ*E* is the applied voltage; in constant-current mode, Δ*E* represents the voltage source for the applied current, known as the equivalent voltage, as shown in Fig. 4.

**Figure 9:**
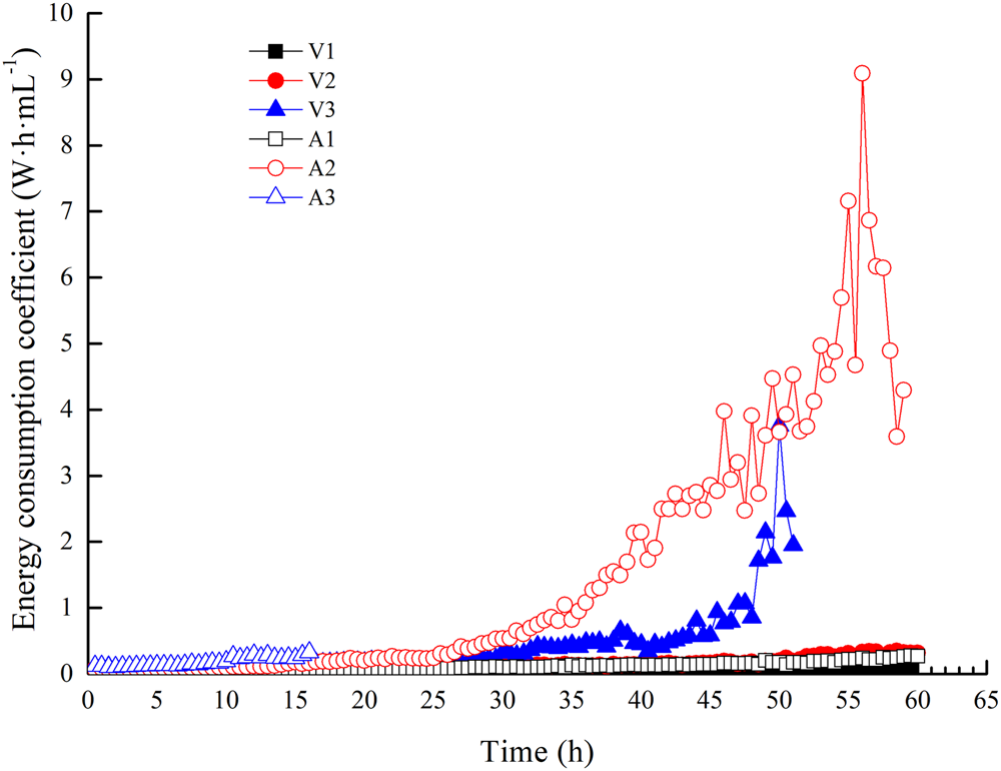
Variations in the energy consumption coefficient with time (overall trend).

**Figure 10:**
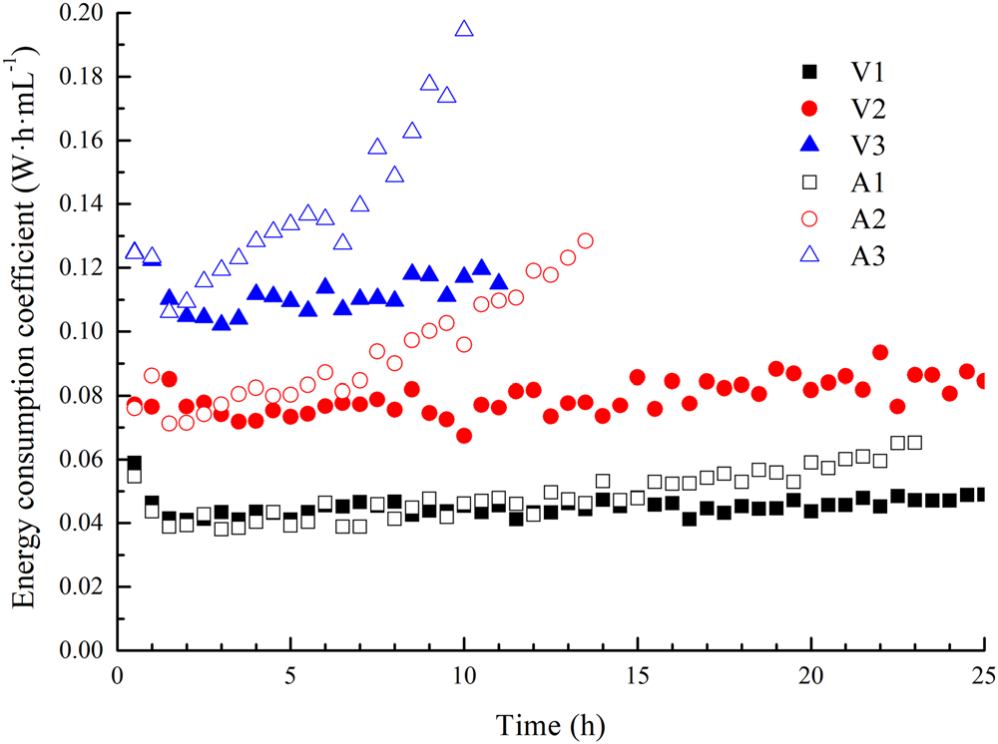
Variations in the energy consumption coefficient with time (forepart).

Figure 9 shows the overall variation trend of the energy consumption coefficient *C* under different working conditions. As previously mentioned, for similar reasons as those described for the measured electro-osmotic transport volume, we focused on the forepart of the electro-osmosis, as shown in Fig. 10.

**Figure 11:**
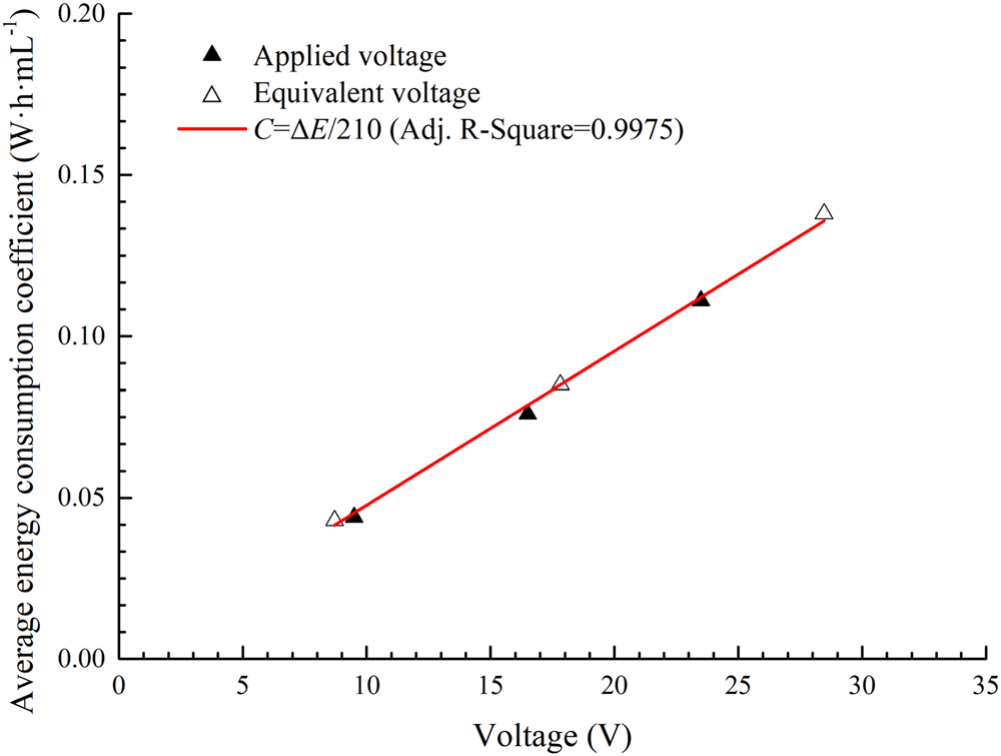
Average energy consumption coefficient with applied/equivalent voltage (forepart).

In the forepart of the electro-osmosis, for constant-voltage mode, the energy consumption coefficient *C* was relatively steady when the applied voltage Δ*E* and the electro-osmotic transport volume *W* remained unchanged; for constant-current mode, the equivalent voltage Δ*E* increased linearly from the initial time, which is the same trend as that observed for the energy consumption coefficient *C*. Tests A1 and V1 had nearly the same energy consumption coefficients because the equivalent voltage of test A1 was close to the applied voltage of test V1, as was the case in tests A2 and V2 and in A3 and V3. As mentioned in section 3.4, the electro-osmotic transport volume *W* was approximately 210 mL·h^−1^·A^−1^ in the early stage (0 ~ 10 h) of all the tests. We also obtained the equivalent voltages of A1~A3: A1 (8.70 V), A2 (17.81 V), A3 (28.46 V), and the average energy consumption coefficients for the forepart of the electro-osmotic tests: V1 (0.044 W·h·mL^−1^), V2 (0.076 W·h·mL^−1^), V3 (0.111 W·h·mL^−1^), A1 (0.043 W·h·mL^−1^), A2 (0.085 W·h·mL^−1^), and A3 (0.138 W·h·mL^−1^). Additionally, we obtained the linear fitting curve of the applied/equivalent voltage and the average energy consumption coefficient by Eq. (7), as shown in Fig. 11.

The above results show that the energy consumption in electro-osmosis is mainly determined by the applied voltage or the equivalent voltage. For the studied type of soil, the electro-osmotic transport volume *W* was determined to be 210 mL·A^−1^·h^−1^. Especially in the forepart of the electro-osmosis, the phenomenon may be different in constant voltage or constant current mode, but the essence is the same. Thus, the power supply mode can be selected according to practical requirements, such as drainage rate control or late-stage enhancement.

### Stage analysis by drainage

**Figure 12:**
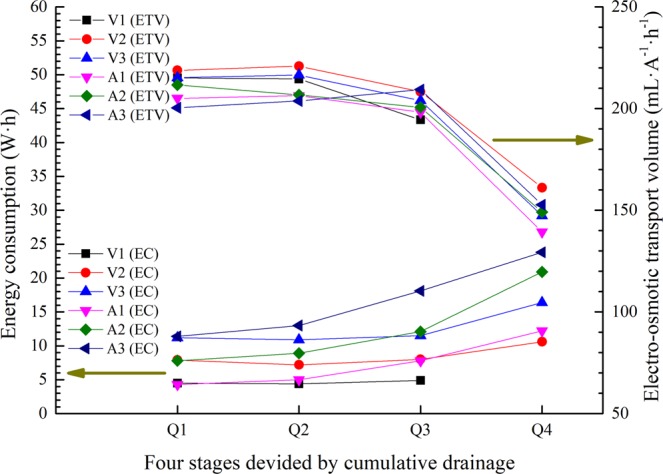
Energy consumption and electro-osmotic transport volume for different stages.

Considering the interaction effect of working modes and time, the different stages of electro-osmosis cannot be exactly reflected by time in this experiment. The lower applied voltage/current tests are still in the early stages with low energy consumption, and the tests with higher applied voltages even approach the end stage. Thus, the electro-osmotic processes are divided into four stages by cumulative drainage: Q1 (0–100 mL), Q2 (100–200 mL), Q3 (200–300 mL) and Q4 (300–400 mL). As the electro-osmotic drainage reaches 400 mL in the tests, the drainage rate is substantially reduced, indicating the electro-osmotic process has developed to the end stage. The distinction of energy consumption and electro-osmotic transport volume in these stages is analysed in different power supply modes, as shown in Fig. 12.

From stages Q1 to Q3, in constant-voltage mode, the energy consumption (EC) remains stable with time and gradually upgrades when the applied voltage is increased. Meanwhile, in constant-current mode, the energy consumption increases with time due to the increase of the equivalent voltage and gradually upgrades when the applied current is increased. In the Q4 stage, the energy consumption for each test increases; the main factor is the shortage of the soil water content. With regard to the electro-osmotic transport volume (ETV), each mode remains stable and shows the same variation tendency. In the Q4 stage, the decreasing of electro-osmotic transport volume value reflects the substantial change of the electrical properties of the soil. This result shows that, for a given type of soil, the value of electro-osmotic transport volume mainly depends on the change of the soil water content rather than on the power supply mode.

## Conclusion

The issue of power supply mode together with the electro-osmosis effect is a current concern in soft soil treatment and reinforcement. The energy consumption and efficiency are considered in practical engineering. In this paper, the effects of different power supply modes, specifically, constant-current modes, were investigated in laboratory tests with an approximately consistent initial electric power and compared with the constant-voltage modes—which are commonly used in practical engineering; the electro-osmotic processes were divided into four stages by equal drainage quantity to obtain the energy consumption and the electro-osmotic transport volume under different working conditions.

The results showed that the electro-osmotic dehydration mechanism in constant-voltage mode and constant-current mode is identical, by which electric energy is converted to water flow. As the electro-osmotic process continued, the electric current decreased in constant-voltage mode, while the electric voltage increased in constant-current mode. To avoid exceeding the rated voltage of the power supply, the adoption of a high current in constant-current modes is not appropriate.

The energy consumption of electro-osmosis is mainly determined by the applied voltage or the equivalent voltage. In constant-voltage mode, the energy consumption coefficient *C* and the energy consumption remain stable as the applied voltage remains constant in the forepart of the electro-osmosis. However, in constant-current mode, from the start, the equivalent voltage Δ*E* increases linearly with the energy consumption coefficient *C*, and the energy consumption increases over time due to the increase in equivalent voltage.

In the main drainage stages of electro-osmosis, the electro-osmotic transport volume of *Ningbo* dredged sediment in different modes is 210 mL·A^−1^·h^−1^, and the electro-osmotic transport volume under different working conditions divided by equal drainage quantity shows that the value of electro-osmotic transport volume depends mainly on the change in soil water content rather than on the power supply mode. The drainage rate in constant-current mode was observed to be relatively steady, maintaining an approximately constant rate until the soil moisture was dramatically reduced. In other words, constant-current mode shows the advantages of being powerful and persistent in electro-osmotic treatment.

## Data Availability

The data used to support the findings of this study are available from the corresponding author upon request.
